# Preparation and Characterization of Self-Healing Polyurethane Powder Coating Using Diels–Alder Reaction

**DOI:** 10.3390/polym13213803

**Published:** 2021-11-03

**Authors:** Negin Farshchi, Michaela Gedan-Smolka, Markus Stommel

**Affiliations:** 1 Leibniz-Institut für Polymerforschung Dresden e.V., 01069 Dresden, Germany; mgedan@ipfdd.de (M.G.-S.); Stommel@ipfdd.de (M.S.); 2 Faculty Mechanical Engineering, Institute of Material Science, Technische Universität Dresden, 01062 Dresden, Germany

**Keywords:** self-healing, uretdione cross-linker, polyurethane, powder coating, reversible bonding, Diels–Alder reaction, film properties

## Abstract

Although powder coating systems offer many environmental, ecological and energy related benefits over liquid based coatings, in the case of uretdione based polyurethane systems, high curing temperature is still an issue. On the other hand, powder coating systems make it possible to reduce the costs and enhance the process of forming complex 3D structures using the deep drawing method by pre-coated metal substrates. During this processing method, there is a probability of micro crack formation in the coated film due to strain impact on the coating layer. A powder coating with self-healing ability is an ultimate solution to face not only this kind of fraction but also any other possible ones (such as defects caused by any impact on film surface during processing, transporting or even service). Here, a single molecule that is prepared via Diels–Alder cycloaddition reaction and retro Diels–Alder cleavage reaction was utilized as a self-healing additive to achieve self-healing ability in the powder coating system that is based on a commercially available uretdione cross-linker and OH-polyester resin. Coatings were prepared through melt mixing of components in a lab mixer, milling, sieving, and then application on the metal substrate through the electrostatic spraying method. To illustrate the role of self-healing additive, various concentrations (4 and 9% wt.) in combination with different curing temperatures (80 °C to 200 °C) were investigated. Both samples containing HA showed self-healing ability at elevated temperature around 120 °C for about 30 min with acceptable roughness and surface properties. Hardness measurement of cured film as well as thermal investigation indicate the chemical reaction of HA in a cross-linked network of cross-linker and resin. In addition, using HA leads to a 40 K drop in curing temperature of the system without using any catalyst. A 2.58% improvement in hardness values at a lower curing temperature and healing time of around 12.5 min at 120 °C to recover 100% of initial scratch (more than 10 cycles) in the sample containing 9% wt. HA was observed.

## 1. Introduction

Self-healing ability in engineering materials has attracted a lot of attention in recent decades since the pioneering study of White et al. [1]. The main idea of self-healing materials is inspired by nature and generally aims at three targets: restoration of mechanical properties of materials, electrical conductivity, or the inhibition of corrosion.

Although many types of research divide self-healable materials into two different groups of the intrinsic and extrinsic self-healing ability [2], until now there is no report of a man-made material which is capable of self-repair without any induced condition such as light, heat, PH, etc.

Many different mechanisms have been applied so far to achieve self-healing ability in materials such as micro- or/and nano-encapsulation (mimicking the bleeding of a small cut) [3] or microvascular networks [4]. In addition, there are various reaction-based strategies such as: sol−gel phase transition [5], click chemistry [6], molecular interdiffusion [7], reversible bond formation through different reactions such as Diels–Alder/retro Diels–Alder (DL/rDL) [8], disulfide links [9], radical recombination [10], anionic reaction [11], etc. 

Considering coating materials, having the self-healing ability is a great advantage not only because it improves the corrosion resistance of the coating but also as it expands its life service. The former benefit also is in favor of the economic and environmental concerns of the coating industry, the same concerns which lead to the development of solvent free powder coatings in the 20th century [12]. Therefore, integrating the self-healing ability into powder coating systems is in favor of powder coating systems goals as well. 

In general, powder coatings are divided into two main groups of thermoplastic and thermoset (cross-linked) based on the starting materials. There are many advantages listed for powder coatings compared to conventional organic coatings [13]. The most important ones are the solvent free production process, the ability of recycling the overspray powder material in industrial lines, economic benefit, and their environmentally-friendly preparation and applications [14]. 

One of the most favorable thermoset powder coating systems is the polyurethane system, due to outstanding overall properties such as weather stability [15]. Isocyanates are a highly reactive component that can react with water or air moisture at room temperature. In order to control this behavior, externally or internally blocked isocyanates, based on uretdione, are highly commercialized. The main disadvantage of uretdione cross-linker is the relatively high curing temperature when used without any catalyst, which leads to different attempts for achieving low temperature curing systems [16,17].

DL/rDL reaction is probably the most well-known chemical reaction used for achieving the self-healing ability in materials [18,19]. Since the first report of thermally reversible polymer networks containing Diels–Alder functionality in 1969 [20], several systems based on this reaction using various functionalities have been investigated and reported [8,21,22]. The Furan–Maleimide system first reported by Chen et al. [23] was formed around 75–80 °C and had nice thermosetting properties upon cooling [24]. In general, this type of reaction can be utilized for the healing of materials in a temperature range of 145 °C for around 25 min. Other researchers also investigated and improved this system to be usable both for higher and lower healing temperatures (time). The reaction between furan and maleimide is usually a coordinated cycloaddition. However, the nucleophilic character of furan derivatives may also lead to an asynchronous state or even a stepwise zwitterionic mechanism in reaction with a p-electron deficient component such as maleimide [25,26]. This concept has been used for most of the materials such as epoxy [27], polyketones [28], polystyrene [29], polyethylene [30], polyamides [31], PMMA [32] and linear polyurethanes [33,34]. 

Material preparation and thermo-reversible DA reaction have already been investigated in polyurethanes [33,35,36,37,38,39,40,41,42]. Polyurethane elastomers have been fabricated using thermally reversible C–ON bonds, and results showed that equilibrium between cross-linking and de-cross-linking of polyurethane chains enables multiple self-healing [43]. By changing the molar ratio of the –NCO to the –OH in the cross-linked polyurethane, Yamaguchi and co-workers reported a self-repairing polyurethane that exhibited a different number of dangling chains [44]. Lin et al. developed a photo-stimulated self-healing polyurethane that contained two dihydroxylcoumarin derivatives, and it was able to regain self-healing behavior by applying UV light repeatedly [45]. A dynamic urea bond was designed by Ying and repaired autonomously at low temperatures [46]. An article by Xu et al. developed a self-healing polyurethane with disulfide links and shape memory effect and scratches almost disappeared in 5 min. After four hours of being kept in healing condition, mechanical properties were nearly restored [47]. Using the Diels–Alder reaction between the furan group and the bismaleimide group, Du prepared a linear self-healing polyurethane [33]. In order to achieve a film with favorable properties, a cross-linked network in the polyurethane system would be essential. On the other hand, using disulfide links in a polyurethane system for coating application might result in change of color (yellowing) of the final product. Although micro capsulation may be widely applicable in self-healing materials, in the case of powder coating the high rate of friction and stress during the processing stage make it hardly acceptable as an option for a self-healing mechanism.

In the case of powder coating, there is an investigation on epoxy systems using reversible reaction to obtain self-healing ability [48]. However, to the best knowledge of the authors, there is no study considering self-healing in uretdione based cross-linkers in polyurethane powder coatings. As the result of several advantages of powder coating systems such as reusable overspray and convenient processing and application method, it is preferred over liquid based polyurethane films. Therefore, here we focused on a cross-linked network rather than linear polyurethane. On the other hand, powder coating polyurethane can be utilized to prepare pre-coated flat sheets, which afterwards form complex 3D structures by deep drawing. Using pre-coated parts leads [49] to saving of more steps in the production line, for example, by enhancing saving the chemicals and water being used in cleaning steps, etc. [49]. Deep drawing process as a high technology to form complex metal based 3D structures (as well as other impacts during process and transport), can cause multiple micro cracks in the film surface, where a film with self-healing ability would be an advantage. 

Considering the high curing temperature/time needed in non-catalyzed uretdione systems (being around 200 °C) and the mentioned advantages of powder coating systems compared to the solvent based coatings, the investigation of self-healing polyurethane coating based on uretdione cross-linker is motivated to extend the final product’s service life. 

In this study, a well-known furan/maleimide DL/rDL reaction were utilized to achieve a self-healing powder coating system based on the commercially available uretdione based cross-linker and polyester resin. Thermo analysis and rheological measurements as well as optical methods were used to prove the self-healing ability and surface properties of the novel PU powder coating basic system, which is also capable of curing at lower temperatures without using any further catalyst. 

## 2. Materials and Methods

The 3-amino-1,2-propanediol (Aldrich, ≥99%), furan (Merck, Darmstadt, Germany stabilized, ≥99%) Maleic anhydride (Merck, Darmstadt, Germany) were used as received. Furfuryl alcohol (Merck, Darmstadt, Germany, ≥98%) was dried with Na_2_SO_4_ and distilled under reduced pressure before use. Ethyl acetate, toluene (Acros organics, Thermo Fisher Scientific, Geel, Belgium, ≥99.8%), ethanol absolute (VWR International, Darmstadt, Germany), petroleum ether (Honeywell, Charlotte, NC, USA), and tetrahydrofuran (Acros, organics, Thermo Fisher Scientific, Geel, Belgium, ≥99.6%) were all analytical grade and used as delivered. The hydroxyl functional polyester resin (Crylcoat 2839-0, Allnex, Frankfurt, Germany) and Vestagon BF1320 uretdione cross-linker (Evonik, Essen, Germany) was used without any further modification. The OH group value of polyester resin and the NCO (lat.) content of the cross-linker was measured to be 50 mg KOH/g and 14.17%, respectively. All the samples were prepared in a way that the NCO: OH ratio was 1:1. Resiflow 88 (Worlée, Hamburg, Germany) and benzoin (Sigma-Aldrich, St. Louis, MO, USA) were used as a flow agent and degassing agent, respectively. 

### 2.1. Synthesis of the DA Adducts

**Figure 1:**
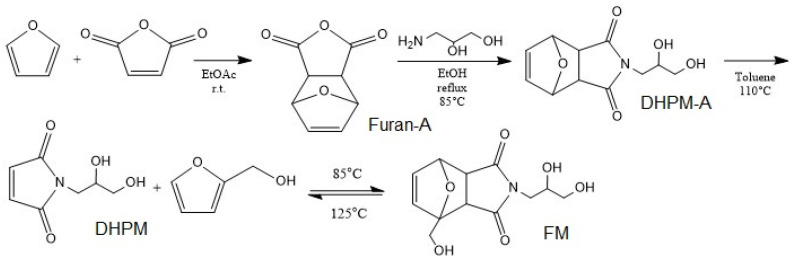
Synthesis of DA healing agent.

The DA adduct was synthesized in the following steps according to the work of Gramlich et al. [50] (Appendix A). It started by the DA reaction between furan and maleic anhydride followed by amine insertion into the product, the resultant underwent a retro DA reaction by refluxing in toluene. Then, at the final step, the healing agent was synthesized by addition of furfuryl alcohol to final product refluxing at 80 °C under N_2_ for 15 h. A mixture of two DA adducts (FM and DHPM-A) in the ratio of 35/65 was utilized as the healing agent (HA) (Figure 1). Nuclear magnetic resonance (NMR) spectra were recorded on a Bruker Avance III 500 spectrometer operating at 500.13 MHz for ^1^H. The measurements were carried out at 30 °C using deuterated dimethyl sulfoxide (DMSO-d_6_) as the solvent, lock, and internal standard (DMSO-d6δ (1H) = 2.50 ppm). 

### 2.2. Preparation of the Powder Coating

**Figure 2:**
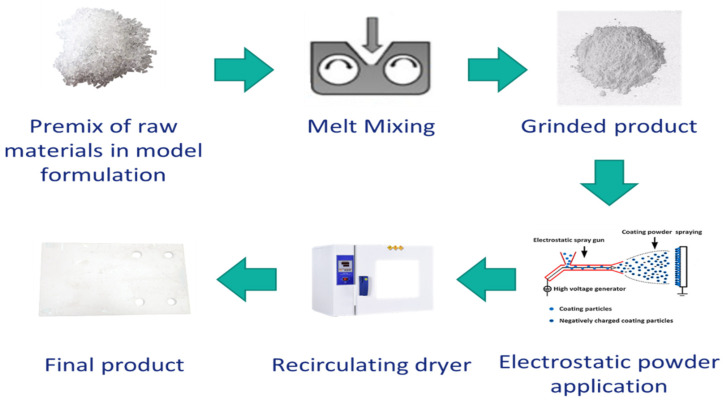
Steps from the preparation of powder coating system to obtain the final cured product.

The powder coating system was prepared through the melt mixing method. First, each component was milled, then pre-mixed to obtain the total formulation, and then the mixture was melt blended via lab mixer (Thermo Fisher Scientific, Karlsruhe, Germany) at a temperature which is well above the glass transition temperature (T_g_) of each component as well as low enough from the reaction temperature. First, a sample was prepared without using HA as the reference (PC1) (at 110 °C, 60 rpm). Then, two different amounts of the HA 4% and 9% wt. were used to prepare PC2 and PC3, respectively (80 °C, 40 rpm). The product was rested for 72 h to complete the relaxation phase. Then, it was milled (IKA A11 basic, IKA-Werke, Staufen Germany), sieved and finally the powders with dimensions ≤63 μm were used for the application. The powder coating was applied on stainless steel (DC04) substrate using an electrostatic gun (GM03, GEMA Switzerland, St. Gallen, Switzerland). The general process to obtain films from powder coating system is depicted in Figure 2.

### 2.3. Film Properties and Characterization

In order to characterize the curing and healing properties of powder coatings the thermal gravimetric analysis (TGA) was performed by a TGA/DSC 3+ device (Mettler Toledo, Greifensee, Switzerland). It was carried out by heating the samples from 30 °C to 800 °C under nitrogen with a heating rate of 10 K/min using around 7 mg of samples in 70 µL Platin Pan. Melting enthalpies were evaluated by integrating areas of melting peaks through using Indium and Zinc for calibration. The differential scanning calorimetry (DSC) was performed on a Discovery 2500 from TA Instruments differential scanning calorimeter. Around 7 mg of each material for all powder samples and HA were heated from 0 °C to 230 °C with a heating rate of 10 °C/min in Tzero Aluminum Pans.

In order to determine the coating properties’ dependence on temperature as well as suitable curing temperature, gradient oven 432 (BYK-Gardner, Wesel, Germany) was used (80, 120, 160 and 200 °C). Then, at each curing temperature, the gloss (Multi Gloss MG 268A, ISO 2813, ISO 7668, ASTM D 523, ASTM D 2457, DIN 67530, JISZ 8741), waviness (wave scan dual BYK-Gardner, IKA A11 basic, Wesel, Germany), as well as impact test (BYK-Gardner, Kugelschlag Prüfer H, DASTM D 2794 at 160 inchxlb) were performed. Additionally, the roughness of the virgin substrate and the coating before and after healing was measured using HOMMEL-ETAMIC W5 (JENOPTIK, Jena, Germany). In addition, the thickness of coating before and after curing was measured by Byko-test 8500b (DIN 50981; 50984).

The rheological measurements were carried out using an ARES rheometer (Rheometric Scientific, New Castle, DE, USA) with parallel plate geometry with a 1 mm gap in oscillating plate–plate mode, at the frequency of 1 Hz on the powder coating material before curing. The samples were compression molded with 25 mm diameter and 1.5 mm thickness prior to testing. Dynamic storage and lost modules (G’ and G”) were measured as a function of temperature between 25 to 135 °C with a heating rate of 2 °C/min, elongation fixed at 1%, which is in viscoelastic range. The torque transducers of the instrument were in range 0.02–2000 g∙cm and the sample temperature was controlled in a closed chamber under nitrogen atmosphere. 

Scanning electron microscopy (SEM) was used to study the coating morphology. Prior to SEM analyses, all samples were coated with a layer of 3 nm Pt. SEM images were obtained by utilizing a Gemini Ultra plus from Carl Zeiss Microscopy GmbH, Jena, Germany, a field emission SEM. In our studies, a SE2-detector (Everhart–Thornley type) was used. Scandisk confocal microscope, µsurf expert, (NanoFocus AG, Germany) was occupied to measure the crack profile before healing as well as to determine the surface profile after healing. Finally, G200 Nanoindenter (KLA, Milpitas, CA, USA) with an XP head was used to investigate the hardness of cured films via depth-sensing nano-indentation.

## 3. Results and Discussion

### 3.1. Thermal Gravimetry Analysis (TGA)

**Figure 3:**
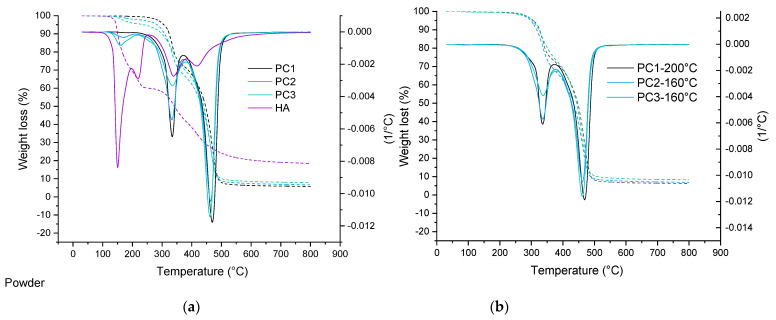
TGA curve comparing thermal degradation behavior of powder materials as well as the healing agent (**a**) and their cured films (**b**).

TGA analysis of basic powder coating samples, as well as cured films, are depicted in Figure 3. In the case of the reference sample (0% wt. HA), an overall mass loss for both the powder and the cured film at 160 °C is the same, around 0.55% wt, while for the samples containing the healing agent, the mass loss is slightly higher for powder samples compared to the cured samples. This is due to the loss of the healing agent that is not incorporated in the cross-linked network, as the temperature is high enough above the DL reaction temperature [51]. Based on the data listed in Table 1, it can be seen that all cure samples show the same behavior. This can also indicate the sufficient cross-link density in samples containing HA. It also can indicate the chemical bonding of the HA into the network [52]. Considering the weight lost at 200 °C, the main question is: what is happening with the HA at this elevated temperature? This question can be answered through a detailed study of the model system in the future (second part).

### 3.2. Differential Scanning Calorimetry (DSC)

**Figure 4:**
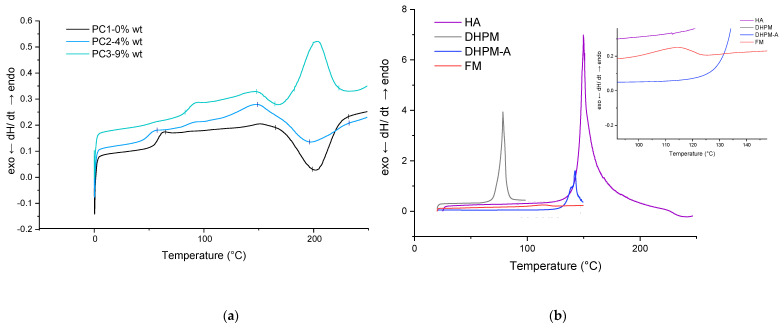
DSC results for first heating scan of powder material: (**a**) reference, PC2 and PC3; (**b**) HA (and each of its components).

The thermal behavior of HA and each sample (powder and film) was determined by DSC measurements under an N2 atmosphere in order to characterize the DA reaction. Figure 4 shows the DSC traces of PC1–0% wt, PC2–4% wt, and PC3–9% wt in the first heating scan before the curing step in addition to the HA DSC curve at the same temperature range (0 to 250 °C). The glass transition temperatures (T_g_) are indicated in Table 2. It is well known from the literature that DA reaction leads to a mixture of tow diastereomers, which are referred to as endo (kinetic product) and the exo (the thermodynamic). During the rDa, the endo unblocks in lower temperature than the exo compound. Several parameters affect the ratio of the endo and exo in the product as well as the deblocking (rDA reaction) temperature (structure, experimental condition, solvent, temperature, etc.). The most important parameter is the substituent electronegative behavior of the furan and maleimide reactants, which not only affects the endo/exo ratio but also the stability of the adduct obtained from the DA reaction [53]. On the other hand, the ratio of exo will increase by increasing the temperature. It can be assumed here as well that the broad endothermic peak above 80 °C until 110 °C corresponds to the endo unblocking reaction (rDA endo), and the other endothermic peak begins at 110 °C until the maximum point of 150 °C corresponding to the exo rDa reaction [54] (in both HA-containing samples, PC2–4% wt and PC3–9% wt), as other researchers also detected this peak attributed to DA reaction [23,24,48].

The addition of the healing agent lowered the T_g_ of the powder system, which is in favor of the self-healing procedure by increasing flexibility. The sharp endo peak around 200 °C is due to melting or/and degradation of HA. In addition, an approximately 18 K shift of the onset of curing temperature is noteworthy (in the absence of any additive or catalyst). 

**Figure 5:**
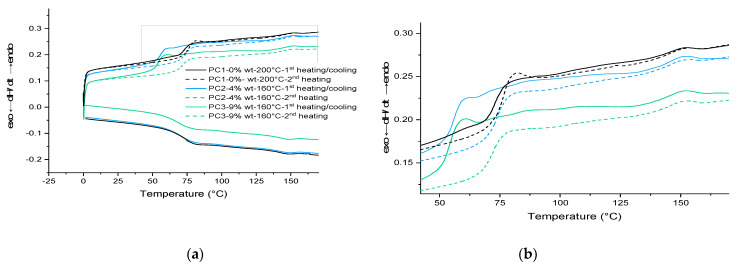
DSC results: (**a**) for cured samples in three heating/cooling/heating cycles; (**b**) magnified part of the curve showing the DL–rDL reaction around 85 °C and 145 °C. PC1, PC2 and PC3 cured at 200 °C and 160 °C for 30 min, respectively.

Then, based on the result achieved from the gradient oven test, each sample containing HA was cured at 160 °C for 30 min in an air-circulating oven. For the formulation without HA, only curing at 200 °C results in a completely cured film. The results for PC1–0% wt. HA cured at 200 °C were used as the reference film properties. The DSC scans for the first heating cycle, cooling and the second heating cycle of cured films are shown in Figure 5. Same as the uncured samples, the broad signal above 80 °C during the first and the second heating scans as well as the same signal below 150 °C during cooling scan correspond to the DA and rDA reaction of HA. This indicates the reproducibility of the healing reaction in both PC2–4% wt. and PC3–9% wt., which can theoretically happen for infinite cycles of healing by heating the damaged samples (above 120 °C) and cooling it down (to room temperature).

Considering the T_g_ values listed in Table 2 and the fact that usually curing a cross-link system leads to increase in the T_g_ value of the system, the behavior of powder containing 9% wt. HA is interesting upon heating and cooling (lower T_g_ value after first heating cycle). However, it is well known that the structure and length of the chains between the cross-linking points can affect this behavior. One reason can be the loss of healing agent as the maximum temperature of measurement (250 °C) is much higher than the decomposition temperature of the HA component. The other reason for the difference between the first and second cycles in the cured samples is the homopolymerization of maleimide around 220 °C [36]. Considering the T_g_ value for the cured film at the first heating cycle (53.49 °C), the idea regarding the loss of the HA at elevated temperatures is more likely. Because of the overlapping of various thermal effects, the thermal behavior of samples needs to be investigated further through model systems in more detail. Results shall be discussed in the next section.

### 3.3. Rheological Measurements

**Figure 6:**
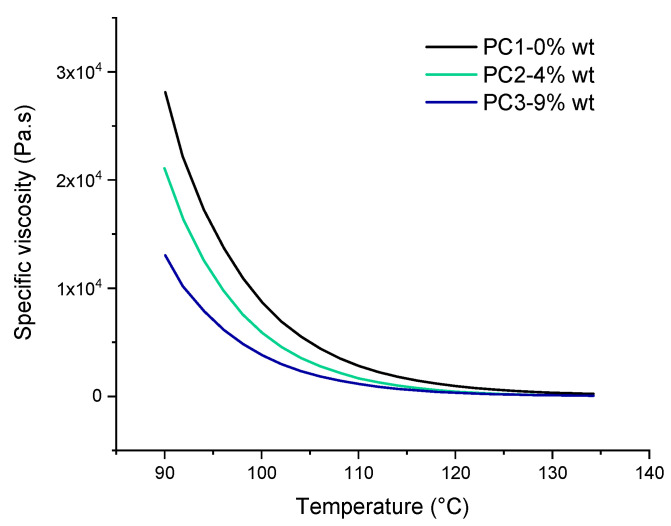
The specific viscosity of uncured basic urethane powders upon heating from 90 to 135 °C.

One of the most important factors that leads to good leveling and film formation of the powder coating system is the significant drop in viscosity of binder upon heating. According to rheological results of the powders before curing, as shown in Figure 6, the addition of DA adduct to the powder coating system has a noteworthy effect on the specific viscosity behavior toward temperature. This reduction in specific viscosity may be due to the fact that at elevated temperature the DL reaction leads to cleavage of polymer chains at the HA molecules cross-linked points leading to shorter chains exhibiting lower viscosity. On the other hand, it could be caused by the usual drop of the melt viscosity with increasing temperature when there is no further reaction, resulting in an increase of the viscosity and/or the solubility of the molecule in the resin-cross-linker melt. The temperature window (90 to 135 °C) is chosen in a way to investigate the effect of DL reaction on rheological behavior of uncured powder material. In addition, it is far below the curing temperature of the powders (160 °C) and also contains the healing temperature (120 °C) used in this study.

**Figure 7:**
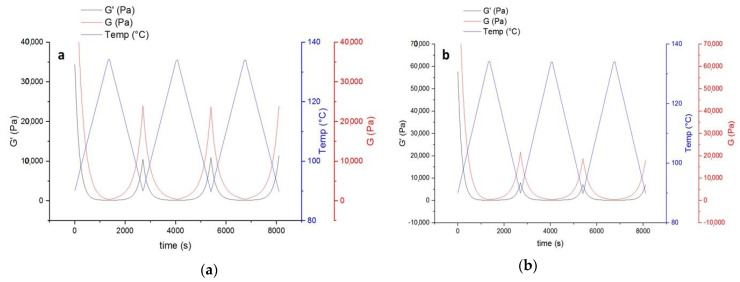
The thermo-reversible behavior of uncured powder samples containing HA based on storage and lost modulus. (**a**) Contains 4% wt. HA, and (**b**) 9% wt. HA.

The decrease of modulus in the first heating step (Figure 7) indicates that introducing HA to the powder coating system leads to improvement of the mobility of molecular chains. This can be in favor of the healing procedure affecting both healing time and temperature. On the other hand, the improved mobility leads to stronger hydrogen bonding with the availability of OH groups in the system (from both functional polyester resin and the HA). Higher values of loss modulus (G’’) compared to storage (G’) indicate higher flexibility of the system, which is again in favor of the healing procedure.

### 3.4. Coating Properties Measurements

In order to investigate the curing temperature effect on the film properties of samples, waviness, gloss, and impact tests were performed on samples prepared by a gradient oven in four different curing temperatures (80 to 200 °C in 40 °C steps). The corresponding results are listed in Table 3. 

As a general fact in coatings, the surface appearance is influenced by brilliance and waviness. Through a wavy pattern of light and dark areas, the multi gloss test measures the specular reflection (gloss) of a surface. In non-metal surfaces such as coatings and plastic, the measurement is angularly dependent, which means the amount of reflected light increases with an increase in the angle of illumination. The results from 60° of illumination may be classified into high gloss (>70 GU), medium gloss (10–70 GU), and low gloss (<10 GU). If the measurement exceeds 70 GU or less than 10 GU, the test setup should be changed to 20° and 85°, respectively [55]. In both samples containing HA, gloss at all temperatures is much higher than in the reference sample. 

Considering the impacts of the test results, it is obvious that addition of HA to the powder coating system leads to a kind of catalytic effect by the HA. Therefore, it was possible to obtain favorable properties from the PC at lower curing temperature (160 °C instead of 200 °C).

### 3.5. Depth-Sensing Nano-Indentation (DSI)

**Figure 8:**
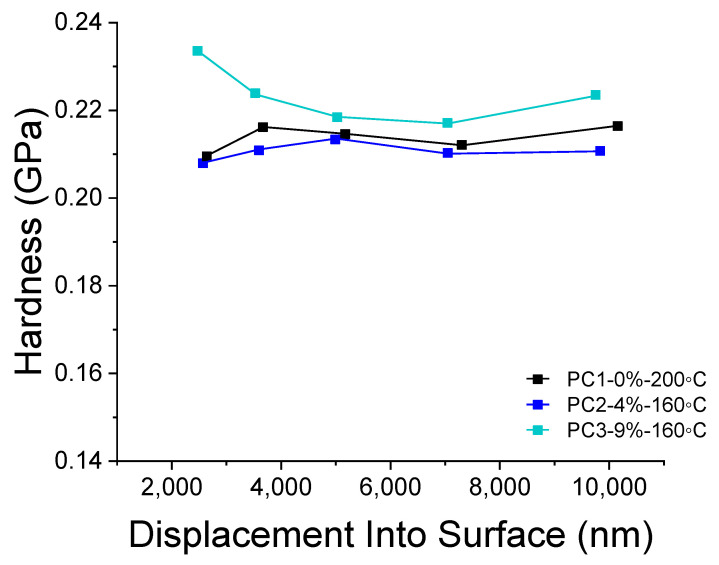
DSI results comparing hardness of PC1 cured at 200 °C and PC2 and PC3 cured at 160 °C.

Depth-sensing nano-indentation (DSI) analysis was carried out to study the hardness of coatings containing various amounts of HA and cured at different temperatures (Figure 8). The hardness of coating can reveal interesting information on the cross-linking of the powders during curing regarding dependence on the temperature. As it is obvious from Figure 8, a curing temperature of 160 °C for samples containing HA resulted in hardness values almost similar to the reference sample cured at 200 °C. The difference in the trend of hardness toward depth of measurement can be due to pile up effect during the indentation. Therefore, it is reasonable to take the values between 3000 nm and 7000 nm (10% of the film thickness) into account. Considering this range (3000 nm to 7000), using 9% wt. of the HA results in a 2.58% increase in the hardness of reference film at an even lower curing temperature (ΔT_curing_ = 40 K). The difference between the reference film and sample containing 4% wt. HA is only 1.29%, which can be considered as almost the same. Doubling the amount of the HA in films cured at the same temperature results in a 3.94% increase of the hardness values. 

### 3.6. Roughness Measurement

**Figure 9:**
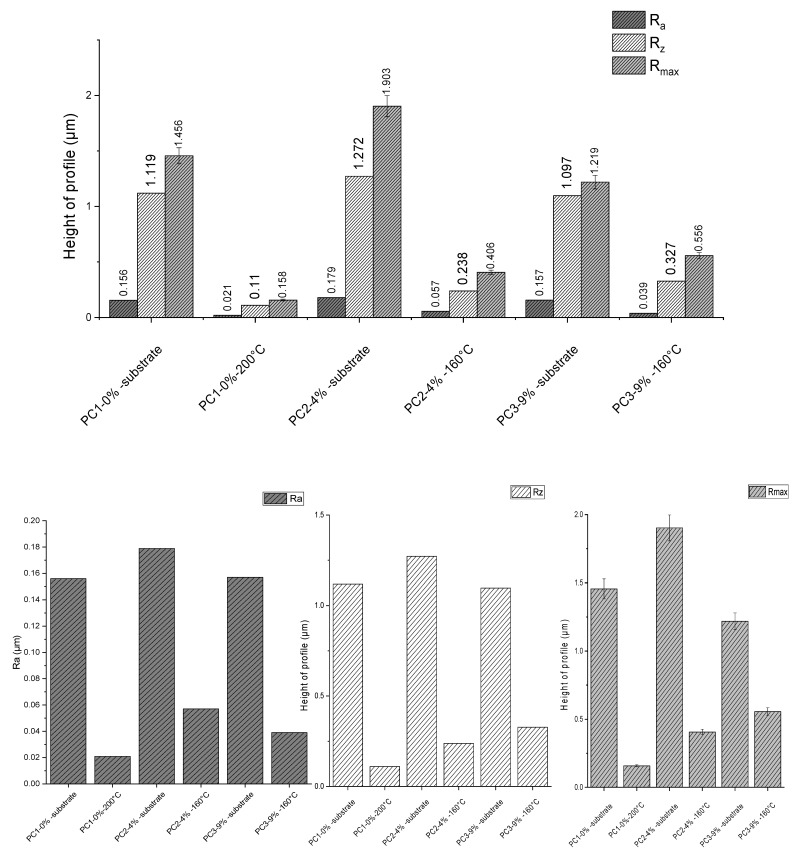
Roughness measurement results for substrate before and after coating for different systems.

The surface roughness of substrates before and after powder coating application was measured and results are depicted in Figure 9. Measurements were repeated five times for each sample. In this graph, Ra is the arithmetical mean deviation, Rz is the mean maximum height of profile and Rmax is the maximum height of profile (according to standard methods DIN EN ISO 4287, DIN EN ISO 4287, DIN 4768:1990). Considering Rz, application of the reference system PC1–0% wt. on substrate resulted in 90.17%, PC2–4% wt. caused 81.29%, and PC3–9% wt. led to a 70.19% reduction. Higher surface roughness using a higher amount of healing agent is clearly due to the presence of HA solid particles in the system compared to the reference system.

### 3.7. Scandisk Confocal Microscope (SCM)

**Figure 10:**
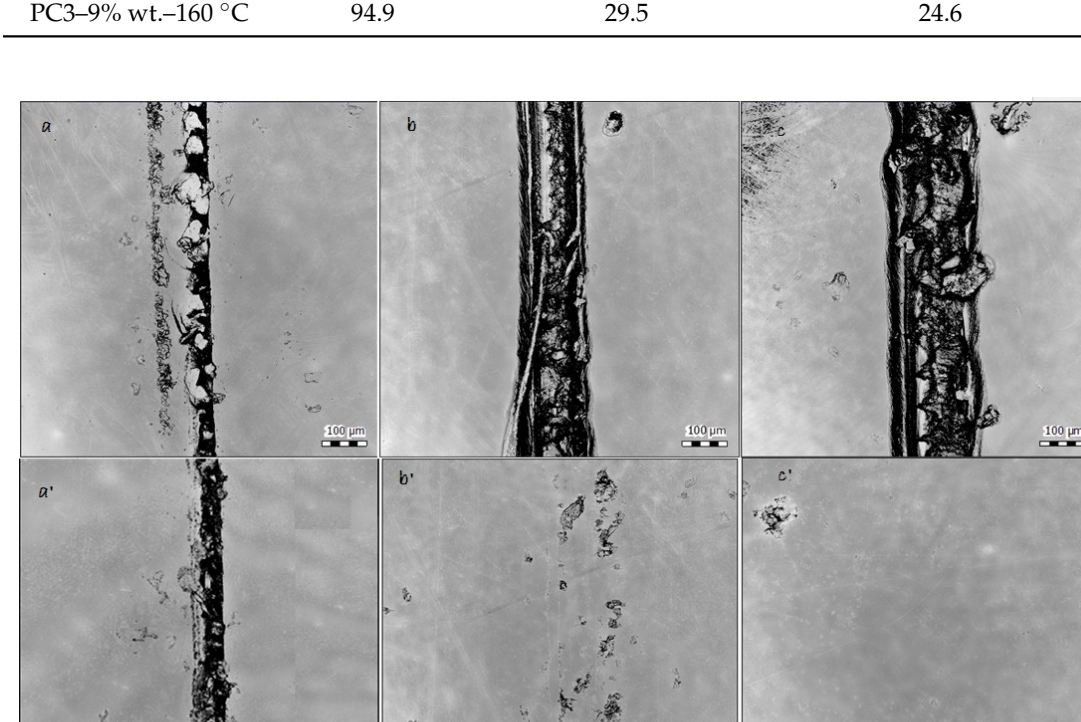
Surface scan of samples before and after being held at 120 °C for 30 min by Scandisk confocal microscope. (**a**) PC1–0% before, (**a’**) PC1–0% after, (**b**) PC2–4%–160 before, (**b’**) PC2–4%–160 after, (**c**) PC3–9%–160 before, and (**c’**) PC3–9%–160 °C after.

To determine the healing ability as well as scratch profile (Table 4), the surfaces were scanned before and after healing of the coatings at 120 °C for 30 min. Results illustrate the healing of the surface in both samples containing HA (Figure 10). Considering Figure 10 b and b’, it can be seen that even using a low amount of the HA as 4% wt. also led to an acceptable amount of the recovery of the surface. Providing higher temperature (or time) did not provide in any better results. However, the healing efficiency in sample PC3–9% wt. at 160 °C is much higher. Here, the recovery of the surface after healing is completely achieved. Moreover, the healing of the scratch at the same place was repeated 10 times for PC3–9% wt. at 160 °C. A complete study of the healing life cycle will be provided in future works. 

### 3.8. Scanning Electron Microscopy (SEM)

**Figure 11:**
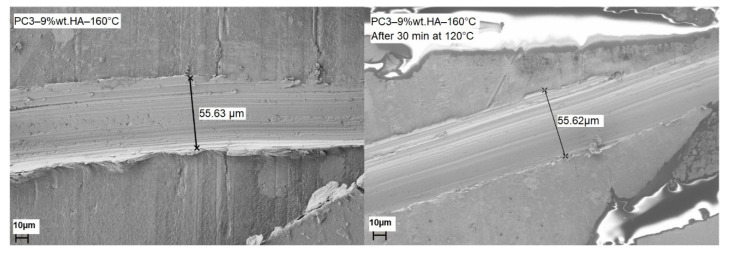
SEM images of PC3–9%–160 °C before and after healing of the scratch by heating sample to 120 °C for 30 min.

SEM images (Figure 11) were obtained to investigate the morphology of the surface as well as the self-healing ability of samples. Considering the results obtained from SCM, PC3–9% wt.–160 °C was used for SEM. In order to capture the self-healing ability of samples a scratch was made using a surgical blade (N11), the width of scratch was measured by SEM to be around 55.62 μm and the length was about 0.5 cm. After measuring the width and morphology of the scratch on the sample, it was annealed at 120 °C for 30 min. The SEM images show the total healing of the scratch and the surrounding area. 

### 3.9. DSC Measurement Coupled with Camera

**Figure 12:**
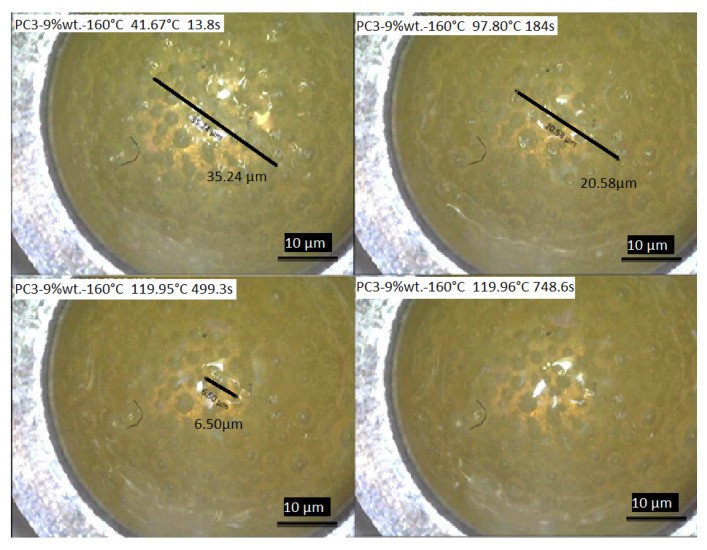
The healing process of the scratch on the film surface of PC3–9% wt.–160 °C recorded during DSC measurements.

For further investigation of the healing process considering healing time, a DSC measurement coupled with a camera was conducted to record the healing of the scratch with initial length of about 35.24 µm and depth of about 15 µm (Figure 12). According to the result, the healing process started by reaching the rDA reaction temperature around 120 ± 0.04 °C in around 4.5 min, and after staying almost 8 min at this temperature the whole defect was healed. 

Considering the heating rate of 10 K/min, which was used to reach the healing isothermal temperature (~120 °C), after only 3 min and reaching ~100 °C, 41.60% of the initial defect healed. By reaching ~120 °C after 8.32 min, 81.55% of the initial scratch was healed. Only 4.15 min at the ~120 °C is needed to achieve 100% recovery of the surface. As this experiment was conducted under air, it gives an acceptable picture of what is happening during the healing process. It should be considered that the thickness of material is much higher compared to the coating samples here (considering the DSC pan size).

## 4. Conclusions

For the first time, a self-healing ability is reported in this study for the polyurethane powder coating systems containing commercial uretdione based cross-linker (BF1320), OH-functionalized polyester resin, and the Diels–Alders adduct as a healing agent. Using HA leads to unlimited cycles of healing (theoretically) as well as relatively lower curing temperature without using any kind of catalyst or additive. In addition, there is no need to synthetize a specific cross-linker or/and resin to achieve self-healing ability. In other words, HA can be used an additive in the powder coating formula. 

Considering the importance of environmentally-friendly characteristics of powder coating systems, this novel self-healing uretdione powder coating system has high capacity for further chemical and physical investigation. The relatively lower curing temperature (40 K drop) is based on specific chemical interaction between uretdione, OH groups of HA and the resin. The 2.58% increase in film hardness with addition of 9% wt. HA at lower curing temperature (ΔT_curing_ = 40 K), in addition to the TGA results for cured film (no sign of HA loss signal), empower this assumption. Further investigation must be carried out in order to demonstrate the exact chemistry of the healing process and the catalysis mechanism using a simple model system.

The repeatability of the healing process was checked through investigation of thermal, physical and rheological properties of powders and coatings via different methods. Between the two investigated amounts of HA, 9% wt. exhibits better self-healing abilities with healing 100% of the initial crack at 120 °C in about 12.5 min as well as 3.94% higher hardness values for films cured at the same temperature, compared to the sample containing only 4% wt. HA. Moreover, it has favorable coating application properties as well. 

Investigation on other parameters which affect the self-healing ability in this system, such as OH:NCO ratio, variant scratch profiles, particle size, the role of addition of different catalysts and additives, and the change of cross-linker type as well as a deeper understanding of its chemical aspects are still under investigation. The results will be published in upcoming reports in the near future.

## Figures and Tables

**Table 1:** TGA data for uncured powder and cured coatings containing 4% wt. and 9% wt. HA and reference sample (cured at 160 °C).

Sample	Healing Agent (% wt.)	Uncured Powder		Cured Film		Difference after Curing
		Weight Loss (%)	Temperature (°C)	Weight Loss (%)	Temperature (°C)	
**PC1**	0%	1	255.16	1	255.66	0.5 ↑
		0.3967	200	0.4000	200	0.0033 ↑
		0.1986	160	0.3230	160	0.1244 ↑
**PC2**	4%	1	166	1	237.33	71.33 ↑
		1.9131	200	0.6458	200	−1.2673 ↓
		0.8036	160	0.4373	160	−0.3663 ↓
**PC3**	9%	1	150.16	1	244.66	94.5 ↑
		4.0425	200	0.5850	200	−3.4575 ↓
		1.7843	160	0.4449	160	−1.3394 ↓

**Table 2:** Glass transition temperature (T_g_) for each sample from DSC measurements for cycles of heating from 0 to 250 °C.

	Tg (°C)	Powder		Cured Film at 160 °C	
Sample		1st Heating	2nd Heating	1st Heating	2nd Heating
PC1–0% wt.		57.27	75.26	73.51	75.85
PC2–4% wt.		48.82	71.73	56.1	74.75
PC3–9% wt.		85.36	32.26	53.49	72.07

**Table 3:** The coating film properties for various curing temperatures at a curing time of 30 min.

Sample	Curing Temperature (°C)	Waviness		Gloss (GU)			Impact Test at 160 (inchxlb) *
		LW	SW	20°	60°	85°	
PC1–0% wt.	80	NM	NM	5.9	1.7	0.4	F
	120	NM	NM	63.9	100	53.8	F
	160	50.9	30.8	109	133	35.2	F
	200	51.6	36.4	80.9	107	76.5	P
PC2–4% wt.	80	NM	NM	17.2	37.9	24.4	F
	120	38	35.9	159	155	97.7	F
	160	22.6	20.9	159	158	95.2	P
	200	20.7	20.7	123	138	93.5	P
PC3–9% wt.	80	NM	NM	17.4	53.6	50.9	F
	120	27.1	17.5	114	125	95.6	F
	160	*26.5*	23.2	126	134	97.6	P
	200	22.5	10.6	80.1	110	88.1	P

NM = Not measureable because of uncured film. * P = pass, F = fail.

**Table 4:** Scratch profile for each sample measured using SCM.

Sample	Width (µm)	Maximum Height (µm)	Mean Height (µm)
PC1–0% wt.–200 °C	94.9	2.02	1.75
PC2–4% wt.–160 °C	94.9	25.0	24.9
PC3–9% wt.–160 °C	94.9	29.5	24.6

## Data Availability

The data presented in this study are available on request from the corresponding author. The data are not publicly available due to the fact that there is no server available so far in the IPF for sharing. It is still under construction.

## Appendix A

In a round bottom flask containing 125 mL ethyl acetate, 92.5 g furan and 100 g maleic anhydride were added. The reaction flask was stirred at room temperature for 24 h, then the colorless crystal product (Furan-A) was removed via suction filtration and dried under vacuum. It was used without any further purification (100 g) and added to a round bottom flask containing 150 mL EtOH, then 56.5 g APO was added to 40 mL EtOH, which was drop wise added to reaction flask with 1.03 molar excess of APO to Furan-A. The mixture refluxed at 85 °C for 5 h and then it was cooled overnight, and the crystallized product (DHPM-A) was removed via suction filtration and dried under vacuum. The white powder DHPM-A (5g) without any further purification was dissolved in 400 mL toluene and was stirred under nitrogen gas flow while refluxing at 115 °C for 12 h. The white solid DHPM was collected after cooling down the flask overnight via suction filtration. The product was washed with petroleum ether and dried under vacuum. At the last step, 8.5 g DHPM was added to 4.9 furfuryl alcohol and were dissolved in 400 mL toluene. The reaction was stirred under nitrogen gas flow while refluxing at 80 °C for 15 h. The white solid FM was collected via suction filtration, washed several times with tetrahydrofuran and dried under vacuum.

The structures and synthesis process in Figure 1 were all confirmed by the 1H-NMR results as below:

4,10-Dioxatricyclo[5.2.1.02,6]dec-8-ene-3,5-dione (Furan-A) 1H-NMR (500 MHz, DMSO-d6) δ: 6.58 (s, 2H, –CHCH=CHCH–), 5.35 (s, 2H, –CHCH=CHCH–), 3.31 (s, 2H, O=CCH). N-(2,3-dihydroxy-propyl)-10-oxa-4-aza-tricyclo[5.2.1.02,6]-dec-8-ene-3,5-dione, (DHPM-A) 1H-NMR (500 MHz, DMSO-d6) δ:6.55 (s, 2H,–CHCH=CHCH–), 5.12 (s, 2H, –CHCH=CHCH–), 4.75 (s, 1H, NCH_2_CHCH_2_OH), 4.55 (s, 1H, NCH_2_CHCH_2_OH), 3.67 (br, 1H, NCH_2_CHCH_2_OH), 3.34 (m, 4H, NCH_2_CHCH_2_OH), 2.92 (s, 2H, O=CCH). N-(2,3-dihydroxy-propyl) maleimide (DHPM), 1H-NMR (500 MHz, DMSO-d6) δ: 7.00 (s, 1H, O=CCH=CHC=O), 4.79 (d, 1H, NCH_2_CHCH_2_OH), 4.583 (t, 1H, NCH_2_CHCH_2_OH), 3.67 (m, 1H, NCH_2_CHCH_2_OH), 3.40 (m, 4H, NCH_2_CHCH_2_OH); Furan–Maleimide adduct (FM), 1H-NMR (500 MHz, DMSO-d6) δ:6.55 (s, 2H, –CHCH=CHCH–), 5.12 (s, 1H, –CHCH=CHCH–), 4.91 (br, 1H, NCH_2_CHCH_2_OH), 4.75 (br, 1H, NCH_2_CHCH_2_OH), 4.54 (br, 1H, –CH_2_OH), 4.05 (d, 2H, –CH_2_OH), 3.68 (m, 1H, NCH_2_CHCH_2_OH), 3.32 (m, 4H, NCH_2_CHCH_2_OH), 3.03 (d,1H, O=CCH), 2.92 (d, 1H, O=CCH).
